# Health care professional’s knowledge and attitude towards cardiopulmonary resuscitation in public sector hospitals of rural Pakistan: A cross sectional study

**DOI:** 10.12669/pjms.42.7.8451

**Published:** 2026-07

**Authors:** Aftab Ahmad Khan, Rubina Barolia, Afshan Nazar, Shafiqa Banu, Aftab Khan

**Affiliations:** 1 Aftab Ahmad Khan, M.Phil. Assistant Professor, Sahara Nursing College, Narowal, Pakistan.; 2 Rubina Barolia, Ph.D. Associate Professor, School of Nursing and Midwifery, Aga Khan University Hospital, Karachi, Pakistan; 3 Afshan Nazar, M.Phil. Sr. Lecturer, School of Nursing and Midwifery, Aga Khan University Hospital, Karachi, Pakistan; 4 Shafiqa Banu, BSCN. Registered Nurse, Aga Khan University Hospital, Karachi, Pakistan; 5 Aftab Khan, M.Phil. Health Research Institute, NIH Research Centre, Khyber Medical College, Peshawar, Pakistan.

**Keywords:** Attitude, Basic life support, Cardiopulmonary resuscitation, Healthcare professionals, Knowledge, Pakistan, Rural health

## Abstract

**Objective:**

To assess the knowledge and attitude of healthcare professionals (HCPs) regarding cardiopulmonary resuscitation (CPR) in rural Pakistan’s public sector hospitals.

**Methodology:**

This cross-sectional study was conducted in Chitral’s selected secondary care hospitals from November 2019 to December 2020. Proportionate sampling enrolled 140 HCPs who were interviewed with a self-administered questionnaire containing 23 knowledge and 21 attitude questions. Data analysis employed SPSS version 21.

**Results:**

Out of 156 participants enrolled, the number of HCPs responded was 140 in which 133 (95%) had a history of (three years) practice in rural areas. 49 HCPs (35%) were BLS certified whereas, 84 HCPs (60%) had attended a course on BLS. Moreover, 99 HCPs (70%) performed CPR more than five times. As per their CPR knowledge, only 3 HCPs (2%) possessed outstanding knowledge while 46 HCPs (33%) proved adequate and 91 HCPs (65%) possessed inadequate CPR knowledge. 105 HCPs (75%) showed positive CPR attitude. Seventy five HCPs (54%) were confused in performing CPR and defibrillation whereas, 72 HCPs (52%) showed poor performance due to lack of knowledge and training. These results hold critical implications for healthcare training programs in rural Pakistan.

**Conclusion:**

CPR knowledge among Chitral HCPs is poor, contrasting with positive attitudes. Recommendations include practical training, BLS recertification, mandated BLS skill, and rigorous monitoring.

## INTRODUCTION

Cardiopulmonary arrest constitutes a persistent medical challenge and a formidable public health issue, with critical implications for mortality rates. Even with advancements in medical interventions, cardiac arrest remains a leading cause of death in numerous countries. For instance, the United States witnesses an annual incidence of 300,000 cases of out-of-hospital cardiac arrest (OHCA), and Europe contends with approximately 350,000 OHCA-related fatalities each year.^1^-^3^ Given the potential occurrence of cardiac arrest both within and outside medical facilities, early diagnosis and swift intervention are pivotal. Addressing this high mortality rate hinges upon healthcare professionals (HCPs) being well-versed in cardiopulmonary resuscitation (CPR) guidelines and equipped with proficient basic life support (BLS) skills. The American Heart Association (AHA) has provided comprehensive guidelines encompassing EMS (Emergency Medical Services) activation, immediate cardiac arrest response, early CPR, and the restoration of spontaneous circulation (ROSC).

In the daily medical landscape, healthcare professionals encounter a myriad of emergencies, necessitating a robust grasp of CPR protocols. However, studies underscore that trainees and junior doctors often lack the expertise to execute CPR effectively.^4^,^5^ This deficit in resuscitation knowledge extends beyond Pakistan, being documented globally in countries such as Turkey, Nepal, Nigeria, India, and Greece.^3^ An integral contributor to this knowledge gap is the omission of CPR training in medical curricula and the absence of ongoing professional development opportunities.^5^,^6^

Pakistan’s healthcare landscape also bears the burden of inadequate CPR and BLS awareness among medical practitioners, nurses, and students.^4^-^6^ The repercussions of this deficiency are far-reaching, potentially leading to medico-legal complications and compromised patient outcomes. Erroneous CPR techniques can even yield counterproductive results, causing CPR-related injuries.^2^ Cardiovascular diseases (CVDs) continue to be a major health burden in Pakistan, with coronary artery disease (CAD) being the leading cause of mortality.

According to the World Health Organization (WHO), heart disease deaths in Pakistan have risen from 19% to approximately 29% of all deaths annually, reflecting a growing trend in cardiovascular morbidity.^1^ The post-pandemic and post-conflict landscape has exacerbated the situation, leading to increased risk factors such as stress, limited healthcare access, and lifestyle changes. The World Health Organization (WHO) further reports that Pakistan faces around 1,100 deaths per day due to CAD, translating to approximately 46 deaths every hour (WHO, 2020). Despite the high burden, research on CVDs in rural areas remains limited, and more attention is needed to address the disparities in health outcomes between urban and rural populations.^7^,^8^

While some studies have assessed CPR awareness among healthcare professionals in urban settings, there exists a dearth of data on CPR knowledge among professionals in rural Pakistan. Moreover, limited studies address the application of updated guidelines and healthcare professionals’ attitudes toward CPR. The absence of comprehensive BLS training and certification, particularly within public sector hospitals, is particularly pronounced in rural locales. Moreover, significant disparities exist in the management of critical care, including early resuscitation measures, between urban and rural areas, as well as between private and public hospitals, with rural and public sector hospitals often facing limitations in resources and training for effective critical care management.^9^ Against this backdrop, the current study endeavors to evaluate the knowledge and attitude of healthcare providers toward CPR in secondary care public sector hospitals in Chitral, Pakistan.

## METHODOLOGY

This quantitative, cross-sectional study in Chitral’s selected public sector secondary care hospitals (DHQ Hospital Chitral, THQ Hospital Tehsil Drosh, Tehsil Booni, Tehsil Garm Chashma) from November 2019 to December 2020.

### Ethical considerations:

Approved by Ethical Review Committee of Agha Khan University Hospital Karachi (Reference No. 2019-2054-7056, Date: December 30, 2019). Participants consented.

Sample size calculated as 156 using WHO calculator. Full-time HCPs with ≥1-year public hospital experience in Chitral were included. Proportionate stratified sampling employed.

### Data collection tool:

Self-administered questionnaire with 23 knowledge and 21 attitude questions. Correct answers scored 1, incorrect or “don’t know” scored 0. Knowledge scores ≤50% marked inadequate. Attitude questionnaire used Likert scale (Disagree=0, Neutral=1, Agree=2). Attitude scores >50% considered positive.

### Statistical analysis:

SPSS version 20.0 used for analysis. Frequencies for categorical, mean and SD for continuous variables. To assess group comparisons, we conducted a T-test and ANOVA. To evaluate the relationship between knowledge and attitude, we used the Chi-square test, with Cramer’s V providing a measure of the effect size. Statistical significance was determined using a threshold of p < 0.05. T-test and ANOVA for group comparisons. Chi-square and Cramer’s V assessed knowledge-attitude relationship. Significance: p<0.05.

## RESULTS

Out of the initial calculated sample size of 156, a total of 140 participants responded, yielding an overall response rate of 90%. Table-I presents a comprehensive overview of the demographic characteristics of the study participants. The participants’ age distribution ranged from 23 to 57 years, with a calculated mean age of 36.2 ± 8.47 years. Notably, 25% of the participants fell within the age bracket of 20-30 years, 44% were aged 31-40 years, 19% were within the 41-50 years range, and 12% were aged above 50 years. In terms of gender distribution, the study comprised 67 (47.85%) male participants and 73 (52.14%) female participants.

**Table-I:** Demographic Characteristics of the Study Participants.

	Frequency (n)	Percentages (%)
Age in years (Mean ± SD)	36.20 ± 8.47	
20 – 30	35	25
31 – 40	62	44
41 – 50	26	18
≥51	17	12
Gender (Male/Female)	67/73	47/52
***Profession***		
Nurse	66	47
Paramedics	44	31
Physicians	30	22
***Qualification***		
RN	48	34
BSCN	18	13
Paramedics	44	31
MBBS	27	18
FCPS	3	2
***Placement***		
DHQ Chitral	80	57
THQ Garm Chashma	21	15
THQ Booni	17	12
THQ Drosh	22	16

Participants were categorized into different professional roles, with 66 (47%) identified as nurses, 30 (22%) as physicians, and 44 (31%) as paramedics. Among the nurses, 72% held a diploma in nursing, while 28% possessed a bachelor’s degree. Among physicians, 90% had completed their MBBS degree, with only 10% holding an FCPS degree. Among the paramedics, all 100% possessed diplomas in paramedical sciences. Geographical distribution highlighted that the majority of participants (80, 57%) were drawn from DHQ Hospital Chitral, followed by 21 (15%) from THQ Hospital Garm Chashma, 17 (12%) from THQ Hospital Booni, and 22 (16%) from THQ Hospital Drosh.

### Professional experience and BLS status:

Regarding professional experience, participants were categorized based on years of clinical practice, falling into four groups: ≤ 2 years, 3-5 years, 6-10 years, and ≥ 11 years. Impressively, 96 (68%) participants had clinical experience exceeding six years, while 31 (22%) possessed 3-5 years of experience, and 13 (9%) had clinical exposure of two years or less. In terms of BLS training, 81 (60%) participants had attended a BLS course. Intriguingly, active BLS certification was held by only 49 (35%) participants, while 91 (65%) were without active BLS accreditation. A noteworthy 132 (94%) participants had experience in practicing CPR, with 82 (58%) performing more than 10 CPR sessions. Detailed data is presented in Table-II.

**Table-II:** Professional Experience and BLS Status of the participants.

	Frequency	Percentage (%)
Total Experience in years (Mean ± SD)	3.04 ± .889	
≤ 2 years	13	9
3 - 5 years	31	22
6 - 10 years	51	36
≥ 11 years	45	32
***Experience in a Public Hospital***		
≤ 2 years	7	5
3 - 5 years	40	28
6 - 10 years	50	35
≥ 11 years	43	30
***Attended BLS Course***		
Yes	81	60
No	59	40
***Current BLS Certification***		
Yes	49	35
No	91	65
***Performed CPR***		
Yes	132	94
No	8	6
***Number of CPR performed***		
≤ 5	40	29
6 - 10	17	12
11 - 20	24	17
≥20	58	41

### CPR knowledge:

The average CPR knowledge score was calculated to be 10.4 ± 3.52. Participants’ responses to knowledge questions indicated an overall correct answer rate of 45%, with correct responses ranging from 9% to 95%. When categorizing knowledge scores, 2% of participants achieved an excellent knowledge score, 33% attained an adequate score, and 65% exhibited an inadequate score. Notably, there were variations in knowledge levels among doctors, with 10% demonstrating excellent knowledge, 50% showing adequate knowledge, and 40% displaying inadequate knowledge. Among nurses, 36% demonstrated adequate knowledge, while 64% showed inadequate knowledge. Among paramedics, 16% had adequate knowledge and 84% exhibited inadequate knowledge. This difference in mean CPR knowledge among doctors, nurses, and paramedics was statistically significant (p-value < 0.001). These findings are succinctly summarized in Table-III.

**Table-III(a):** Overall Knowledge Scores among HCPs (n=140).

Knowledge Score	Frequency (%)			P value
	Excellent	Adequate	Inadequate	
Physicians	3(10%)	15 (50%)	12 (40%)	<0.001*
Nurses	0	24 (36%)	42 (64%)	
Paramedics	0	6 (16%)	37 (84%)	

P-value was calculated using Fisher Exact test, with significance level at p<0.05.

**Table-III(b):** Selected CPR knowledge item correct-response rates by profession (n=140).

Knowledge Item	Physicians n(%)	Nurses n(%)	Paramedics n(%)	Total n(%)	p	V
CPR abbreviation recognition	31(88%)	47(77%)	30(70%)	109(78%)	0.138	0.145
Compression: ventilation ratio (adult)	24(68%)	40(65%)	21(48%)	85(61%)	0.088	0.141
Defibrillation indications	17(48%)	27(44%)	19(44%)	63(45%)	0.235	0.073
First action: alone with unresponsive victim	12(34%)	19(31%)	9(20%)	40(29%)	0.372	0.085
First intervention for pulseless patient	15(43%)	18(30%)	11(25%)	44(31%)	0.084	0.135

Chi-square test, df=2. No item reached p<0.05, indicating knowledge gaps are uniformly distributed across all professional cadres.

### Attitude toward CPR:

Participants demonstrated an overall positive attitude toward CPR, with a mean attitude score of 12.6 ± 3.2. A considerable proportion of participants (105, 75%) exhibited a positive attitude, whereas a smaller percentage (35, 25%) demonstrated a negative attitude. When analyzing participants’ willingness and confidence to initiate CPR, an encouraging trend emerged. Physicians and nurses expressed higher confidence in initiating CPR and defibrillation compared to paramedics. The willingness to perform mouth-to-mouth ventilation was particularly evident among physicians and paramedics, whereas nurses showed relatively lower willingness. When it came to the influence of the victim’s gender on CPR initiation, 78 (57%) participants stated that the gender of the victim did not impact their CPR performance. These findings are elaborated upon in Table-IV.

**Table-IV:** Reported positive attitude towards CPR among physicians, nurses and paramedics (n=140).

Questions	Physicians	Nurses	Paramedics	Overall	P-Value
***I can perform CPR on my own.***					
	23 (66%)	40 (66%)	15 (36%)	78 (57%)	.009
***I know how to defibrillate.***					
	15 (43%)	33 (54%)	13 (31%)	60 (43%)	.001
***I would perform mouth to mouth ventilation irrespective of patient age and gender.***					
	27 (77%)	30 (49%)	24 (57%)	81 (59%)	.01
***I think lack of self-confidence influence my performance during CPR.***					
	16 (46%)	31 (51%)	28 (67%)	75 (54%)	.011
***I think gender of the victim affects me in initiating chest compression.***					
	6 (17%)	13 (21%)	15 (36%)	34 (25%)	.05

P-value by Chi-Square test or Fisher Exact Test. P-value < 0.05 * is statistically significant.

### Association between CPR knowledge and attitude:

An intriguing association emerged between CPR knowledge and attitude. Participants who demonstrated excellent CPR knowledge scores consistently held positive attitudes. The statistical analysis corroborated this association, with a significant p-value of 0.03. The Cramer’s V value of 0.304 further emphasized a moderately strong association between CPR knowledge and attitude. These findings are elaborated upon in Table-V.

**Table-V:** Association between CPR Knowledge and Attitude Score (n=140).

Variables	Attitude category (n=140)		P-value
Knowledge category	Positive Attitude	Negative Attitude	Exact Sig
Excellent	3	0	0.036*
Adequate	25	15	Cramer’s V
Inadequate	42	55	.304

P-value was calculated using Fisher exact test, with significance level at p<0.05.

## DISCUSSION

In our study, 140 HCPs from rural public hospitals were surveyed to find the overall knowledge, awareness, and attitude towards BLS and/or CPR. Their mean age was 36.2 ± 8.6 years, with physicians and paramedics being older on average than nurses. Notably, the majority fell within the 30 to 40 age group, aligning with similar studies in Pakistan, Bangladesh, and Ethiopia.^6^,^10^,^11^ The nursing workforce largely consisted of diploma holders (72%) and bachelor’s degree holders (28%). Remarkably, the low ratio of graduate nurses reflects historical educational trends in Pakistan, necessitating a shift towards higher qualifications to improve patient outcomes.

Analysis of clinical experience revealed a well-experienced workforce, with 65% having more than six years of experience. Despite this, a concerning finding was the low proportion of healthcare professionals (35%) with current Basic Life Support (BLS) certification, emphasizing the need for regular training updates. Moreover, while a substantial number performed CPR, formal training was lacking, raising concerns about the quality of interventions during cardiac arrests. This mirrors patterns in Pakistani healthcare settings where a considerable number lacked current CPR certifications.^6^,^7^,^12^

The analysis further depicted that those participants who had fewer years of experience in the public sector hospitals had a higher mean knowledge score 11.7 ± 2.6, whereas, participants having more experience in the public hospitals had the least mean knowledge scores 9.6 ± 4.3. The possible reason could be the lack of continuous medical education, spot assessment, and mandatory certification in the study settings; thus the knowledge was depleting with the increasing years of experience. The finding was consistent with the study done in Pakistan, in which they reported that participants having less experience had higher mean CPR knowledge, as compared to those having higher clinical experiences. This difference was statistically significant, p=0.005, indicating an urgent need for mandatory periodic BLS recertification to prevent progressive knowledge decay with increasing years of tenure.^13^-^15^

In terms of CPR knowledge, the study unveiled significant deficits among healthcare professionals, with 65% demonstrating inadequate knowledge. This is consistent with findings in Pakistan and various other countries, underscoring the critical need for structured training programs.^16^-^18^ This gap in their knowledge, poses a serious concern, as sound BLS knowledge is mandatory for HCPs, as they are expected to encounter cardiac arrest patients on a routine basis. Significant variations in CPR knowledge emerged among doctors, nurses, and paramedics. Physicians fared better, with 60% exhibiting acceptable knowledge scores, compared to 36% among nurses and 16% among paramedics. Recent finding from Lahore Pakistan reported that 83% of HCPs had a poor CPR knowledge. Studies from LMICs including Botswana, India, and Pakistan reported that inadequate knowledge among HCPs was attributed to the lack of routine CPR in the training for HCPs.^11^,^19^,^20^

Despite knowledge gaps, HCPs exhibited positive attitudes towards CPR, echoing findings in India, Botswana, Qatar, Pakistan underscoring the willingness to engage in lifesaving interventions.^13^,^16^,^17^, ^21^ Physicians exhibited the highest attitude scores, reflecting their confidence and willingness to engage in resuscitative efforts. However, concerns arose regarding confidence levels, particularly among paramedics, highlighting the need for targeted training interventions to boost self-efficacy. This novel finding emphasizes the importance of tailored training interventions to address specific needs of all HCPs and enhance the overall readiness to respond effectively to cardiac emergencies.

This study unveils a unique cultural barrier, such as gender preferences, emerged as significant factors affecting CPR practices within the context of rural healthcare in Pakistan, where gender-specific reservations hinder the willingness of healthcare professionals to engage fully in CPR interventions, with females exhibiting reluctance towards mouth-to-mouth ventilation with opposite genders, and males hesitating to administer chest compressions in similar scenarios. A single study in Bangladesh reported gender based barriers but no significant association was found in studies conducted in Pakistan. Importantly, this bidirectional pattern, females reluctant to perform mouth-to-mouth ventilation on male patients, and males hesitant to perform chest compressions on female patients, was observed in more than 60% of both groups, representing a novel finding not previously documented in Pakistani literature and highlighting the need for culturally sensitive CPR training curricula.^18^,^21^,^22^

This finding underscores the importance of culturally sensitive CPR training programs tailored to address gender-specific concerns, ultimately optimizing the readiness of healthcare providers to perform lifesaving interventions in diverse socio-cultural contexts. Notably, attitudes were significantly associated with profession, education, and CPR training, emphasizing the role of education and training in shaping attitudes towards resuscitation efforts.^21^,^23^ A positive correlation between knowledge and attitude towards CPR was observed, highlighting the interdependence of knowledge acquisition and favorable attitudes towards resuscitation efforts. Furthermore, demographic variables such as profession, education, experience, and CPR training significantly influenced both knowledge and attitude scores, emphasizing the importance of targeted interventions to enhance CPR awareness and confidence among HCPs.

### Limitations

While the findings from this study provide valuable insights, their generalizability to other healthcare settings may be limited. The relatively small sample size and the fact that the study was conducted in only one district suggest that the results might not fully represent the diversity of healthcare contexts across the country. However, in settings with similar demographic, cultural, and healthcare characteristics, it is possible that similar trends could be observed. Further studies with larger and more diverse samples across multiple regions would be necessary to confirm the broader applicability of these findings.

## CONCLUSION

This study highlights critical knowledge gaps and attitudinal considerations among healthcare professionals regarding CPR in rural hospital settings. Addressing these gaps through targeted training initiatives and cultural awareness programs is imperative to enhance emergency response outcomes and improve patient care. Future research should focus on evaluating the effectiveness of educational interventions and exploring innovative approaches to CPR training tailored to the unique needs of healthcare professionals in rural contexts.

### Recommendations

The findings advocate for the implementation of targeted training programs and continuous educational initiatives aimed at enhancing CPR proficiency among healthcare providers. Addressing these gaps not only improves emergency response outcomes but also significantly elevates the quality of patient care within rural healthcare contexts. Further studies are required to determine the factors effecting CPR knowledge gap among healthcare professionals in rural context of Pakistan.
